# Effects of High Dietary Carbohydrate and Lipid Intake on the Lifespan of *C. elegans*

**DOI:** 10.3390/cells10092359

**Published:** 2021-09-08

**Authors:** Berenice Franco-Juárez, Saúl Gómez-Manzo, Beatriz Hernández-Ochoa, Noemi Cárdenas-Rodríguez, Roberto Arreguin-Espinosa, Verónica Pérez de la Cruz, Daniel Ortega-Cuellar

**Affiliations:** 1 Departamento de Neurodesarrollo y Fisiología, División de Neurociencias, Instituto de Fisiología Celular, UNAM, Ciudad de México 04510, Mexico; franco_beju@hotmail.com; 2 Laboratorio de Bioquímica Genética, Instituto Nacional de Pediatría, Secretaría de Salud, Ciudad de México 04530, Mexico; saulmanzo@ciencias.unam.mx; 3 Laboratorio de Inmunoquímica, Hospital Infantil de México Federico Gómez, Secretaría de Salud, Ciudad de México 06720, Mexico; beatrizhb_16@comunidad.unam.mx; 4 Laboratorio de Neurociencias, Instituto Nacional de Pediatría, Secretaría de Salud, Ciudad de México 04530, Mexico; noemicr2001@yahoo.com.mx; 5 Departamento de Química de Biomacromoléculas, Instituto de Química, Universidad Nacional Autónoma de México, Ciudad de México 04510, Mexico; arrespin@unam.mx; 6 Neurochemistry and Behavior Laboratory, National Institute of Neurology and Neurosurgery “Manuel Velasco Suárez”, Ciudad de México 14269, Mexico; veped@yahoo.com.mx; 7 Laboratorio de Nutrición Experimental, Instituto Nacional de Pediatría, Secretaría de Salud, Ciudad de México 04530, Mexico

**Keywords:** *C. elegans*, lifespan, carbohydrate, lipids, transcription factors and metabolism

## Abstract

Health and lifespan are influenced by dietary nutrients, whose balance is dependent on the supply or demand of each organism. Many studies have shown that an increased carbohydrate–lipid intake plays a critical role in metabolic dysregulation, which impacts longevity. *Caenorhabditis elegans* has been successfully used as an in vivo model to study the effects of several factors, such as genetic, environmental, diet, and lifestyle factors, on the molecular mechanisms that have been linked to healthspan, lifespan, and the aging process. There is evidence showing the causative effects of high glucose on lifespan in different diabetic models; however, the precise biological mechanisms affected by dietary nutrients, specifically carbohydrates and lipids, as well as their links with lifespan and longevity, remain unknown. Here, we provide an overview of the deleterious effects caused by high-carbohydrate and high-lipid diets, as well as the molecular signals that affect the lifespan of *C. elegans*; thus, understanding the detailed molecular mechanisms of high-glucose- and lipid-induced changes in whole organisms would allow the targeting of key regulatory factors to ameliorate metabolic disorders and age-related diseases.

## 1. Introduction

It is well known that chronic exposure to elevated glucose or lipids through one’s diet promotes the development of metabolic diseases such as obesity and type 2 diabetes, which may impinge on health-related quality of life. Nowadays, both pathologies have substantial impacts on public health and safety because they exacerbate the burden on health services. An increasing number of studies have demonstrated that metabolic diseases cause metabolic dysregulation, which affects energy homeostasis [1,2]. Although many advances have been made in understanding the pathophysiology of energy-related diseases, their prevalence continues to increase, in part because of lifestyle changes and increased overall life expectancy.

Carbohydrates and lipids are biological molecules that are the main energy sources for all life, since their metabolism impacts organismal maintenance, growth, and reproduction [3]; however, overnutrition of carbohydrates and lipids may induce perturbations in whole-body metabolism, which could trigger metabolic-related human diseases such as insulin resistance, metabolic syndrome, and type 2 diabetes [4,5]. It is now well established that excessive lipid and carbohydrate intake can lead to the dysregulation of various metabolic pathways, although the entire molecular mechanisms of their regulation are not yet fully understood [6,7].

**Figure 1:**
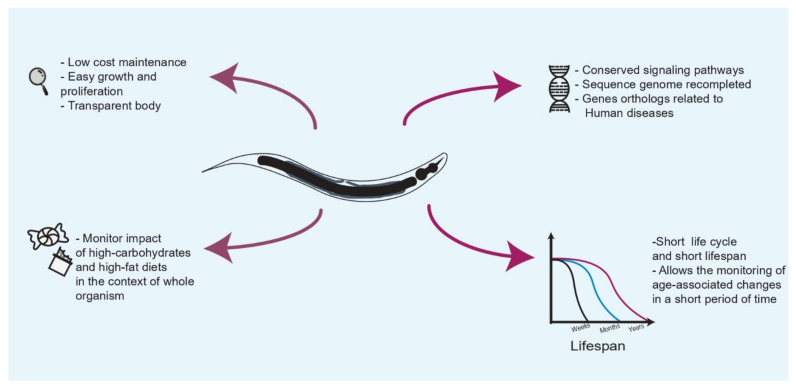
*Caenorhabditis elegans* as a model organism to study the effects of high-carbohydrate and high-fat diets on the lifespan. *C. elegans* offers many technical and biological advantages, since it has been shown that develops many of the cellular and molecular alterations reported in humans caused by overconsumption of sugars and lipids.

The free-living and non-pathogen nematode *Caenorhabditis elegans* has been considered for a long time as a model organism for the study of human diseases because of its highest conservation of signaling pathways and genes responsible for the development of many illnesses, including metabolic disorders [8,9]. Interestingly, the genome of *C. elegans* was the first multicellular genome to be almost completely sequenced, and although its genome has been recompleted in recent years, to date more than 100 genes that have human orthologs have been identified, many of which are related to numerous human disorders [10,11,12]. In particular, *C. elegans* offers many technical and biological advantages, since its maintenance in the laboratory is very cheap and easy and experiments do not involve ethical concerns. Its transparent body allows the identification and monitoring of individual cells and for examination of its development. With a few specimens it is possible to obtain large nematode populations in a short period of time, which are capable of performing many biological and experimental replicates. Perhaps one of the most interestingly advantages of using *C. elegans* as a research model to investigate the detrimental effects of high-glucose and high-fat diets on lifespan is its short lifespan, as the nematode lives for about three weeks, thereby decreasing the time spent assessing the impacts of high consumption of glucose and lipids over the lifespan, in contrast to other multicellular organisms that can live for several months or years. Notably, the influence of such diets on healthspan can be analyzed at the organismal level by assessing changes in its motility, body integrity, fertility, and body appearance [13]. Moreover, it has been shown that carbohydrate and lipid overload trigger deleterious effects similar to those described in humans; for example, glucose-enriched diets in *C. elegans* reduce its lifespan by affecting several components of lipid and glucose metabolism, leading to abnormal accumulation of intermediate metabolites that may have toxic effects on the nematode [14,15]. As such, the worm *C. elegans* represents a relevant model for elucidating the regulatory metabolic regulation mechanisms related to carbohydrate and lipid metabolism and can improve the understanding of nutrition-related factors and their impacts on the signaling pathways that are responsible for the development of metabolic disorders, which in consequence modify the human lifespan (Figure 1).

## 2. Impacts of Carbohydrate Metabolism on Lifespan

In general, the energy used by organisms is predominantly derived from the oxidation of carbohydrates [16], whose regulation is mediated by several genetic pathways to modulate their uptake, storage, and utilization, which have obvious influences on metabolism and can also impact the lifespan.

An increase in the consumption of carbohydrates and low physical activity lead to overweight, which is associated with long-term conditions such as diabetes, cardiovascular disease, and many types of cancers, with obesity being a risk factor for developing several chronic diseases, in addition to having been proposed as an aging accelerator because of its potential to induce many of the hallmarks of aging (genome instability, telomere attrition, epigenetic alterations, loss of proteostasis, deregulated nutrient sensing, and mitochondrial dysfunction) [17]; nevertheless, lifestyle modifications can help avoid or decrease such unhealthy disorders [18]. It has been shown that low-carbohydrate diets are effective for weight loss and improving high-density lipoprotein cholesterol (HDL) and triglycerides profiles [19]; moreover, glycolytic inhibition has been suggested as an effective strategy because of its anti-aging and pro-longevity effects.

Although the roles of dietary carbohydrates in the human lifespan are unclear, the reduction in whole-body carbohydrate metabolism is associated with the elderly, and substantial evidence has shown that glucose tolerance decreases with advancing age; in fact, the decline begins in the third or fourth decade of life and is progressive throughout the entire adult lifespan [20]. These changes can increase the risk of insulin resistance and type 2 diabetes during aging, thereby decreasing the human lifespan.

To increase the knowledge about the cellular damage induced by these conditions, high-carbohydrate intake studies in *C. elegans* have been conducted in high-glucose diets that mimic the high-carbohydrate diets consumed by humans. One of the most intriguing results is the reduction of its lifespan and changes in several metabolic regulators that contribute to it exerting deleterious effects by which glucose decreases lifespan [21].

### 2.1. Glucose Breakdown

For the metabolization of glucose to begin, it needs to be transported inside the cell across the cellular membrane via the glucose transporters, of which there are two main types: sodium–glucose-linked transporters (SGLTs) and facilitated diffusion glucose transporters (GLUTs) [22]. In mammals, the mechanism for glucose transport is primarily mediated by the insulin-sensitive facilitative glucose transporter GLUT-4 [23,24], whereas in *C. elegans* more studies are required to better understand the mechanism of sugar transport. Genomic analyses have shown that the *C. elegans* genome encodes nine high-sequence homologies to human GLUTs, where FGT-1 is the principal glucose transporter, which is ubiquitously expressed in the nematode [25,26]. Interestingly, as with mammals, it appears that FGT-1-mediated glucose uptake is also dependent on insulin or IGF-like signaling (IIS), since the mutations of key proteins such as DAF-2 (homolog to insulin and IGF-I receptor), AGE-1 (homolog to phosphoinositide-3-OH kinase, PI3K), and AKT1/2 (homolog, or protein kinase B/AKT-) impair glucose uptake due to diminished FGT-1 expression; moreover, FGT-1 activity can also be modulated by O-linked glycosylation, similar to mammalian GLUT1, because a mutation in the gene that encodes O-GlcNAcase (*oga-1* mutant strain) decreases glucose uptake by diminishing FGT-1 expression and function [25]. Nevertheless, further studies are required to investigate the fine-tuning mechanism of FGT-1 regulation. Due to the relationship between FGT-1 and IIS signaling, and given that reduced IIS increased *C. elegans* lifespan, Feng et al. investigated knockdown of FGT-1 expression and found that inhibition of FGT-1 leads to an extension of its lifespan in a similar fashion to glucose restriction, suggesting that inhibiting glucose transporter FGT-1 could counteract the adverse effects of glucose intake (Table 1) [27]. Interestingly, opposite results were seen for Glut-4 in mammals, because its reduced expression in skeletal muscle during aging has a negative impact on health via producing insulin resistance [28,29].

**Figure 2:**
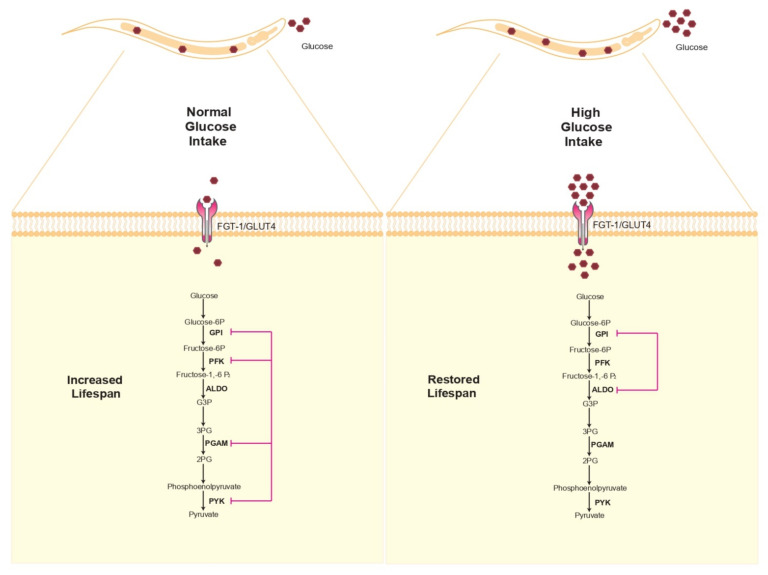
Effects of high glucose in *C. elegans*. Under standard feeding conditions, genetic inhibition of several glycolytic enzymes increases lifespan (left). Exposure to high dietary glucose reduces the lifespan; however, genetic blocks of GPI and ALDO glycolytic genes protect against high-glucose-induced shortened lifespan. GPI, glucose phosphate isomerase; PFK, phosphofructokinase; ALDO, fructose-1,6-bisphosphate aldolase; PGAM, phosphoglycerate mutase; PYK, pyruvate kinase; FGT-1, facilitated glucose transporter -1).

After glucose uptake, glycolysis is active in breaking down glucose and extracting energy or storing it in glycogen form. An analysis to determine whether glycolytic genes are associated with lifespan in *C. elegans* showed that of the three major irreversible regulatory enzymes of glycolysis, hexokinase (HXs), phosphofructokinase (PFK), and pyruvate kinase (PYK-1,2)-, both PFK and PYK knockdown extended the lifespan of *C. elegans* [21,30,31]; a similar effect was seen for glucose phosphate isomerase (GPI) and phosphoglycerate mutase (PGAM), which are also glycolytic enzymes (Figure 2) [32,33]. This effect was probably due to the blockade of glucose metabolism, which likely altered the metabolic flux, reducing mitochondrial respiration or ATP synthesis, thereby increasing the lifespan, similarly to caloric restriction, although we cannot disregard that other pathways may be implicated in increasing lifespan. Glycolysis exerts a particularly strong negative influence on healthy aging, with evidence suggesting that glucose metabolism shifts from aerobic to anaerobic with age and that persistent glycolysis would increase the intracellular load of glycating agents (methylglyoxal, a highly toxic glycating agent) and increase ROS generation, which provokes negative effects on mitochondria [34], contrary to diminishing glycolytic flux through fasting, which suppresses glycolysis, thereby decreasing methylglyoxal production.

A high-glucose diet is considered a determinant factor for the development of obesity, type 2 diabetes, and cardiovascular diseases in humans [35,36]. To increase the knowledge about the cellular damage that provokes these conditions, studies in *C. elegans* have been undertaken using similar high-glucose diets. The results showed that the reduction of the lifespan of *C. elegans* involved many of the enzymes related to glycolysis [37]. To analyze whether enzymes of the glycolytic pathway are involved in the diminished lifespan of nematodes, Lee et al. grew worms on a high-glucose diet and inhibited each step of the glycolytic pathway using RNAi. They found that genetic inhibition of glucose GPI or fructose-1,6-bisphosphate aldolase (ALDO 1,2) is capable of suppressing glucose toxicity and restores the lifespan (Figure 2). Interestingly, the deleterious effects are in part due to the blockade of these enzymes because its absence may reduce glycolytic key intermediaries, such as dihydroxyacetone phosphate [38]. Given the above evidence, we speculate that high glucose levels and high glycolytic activity negatively influence the general lifespan of *C. elegans*.

### 2.2. Lifespan-Related Pathways Affected by High Glucose

Several studies have found a strong correlation between the intake of excessive dietary carbohydrates and metabolic disorders that consequently diminish health and lifespan. There are several signaling pathways by which elevated glucose exacerbates its toxic effects and negatively influences the lifespan of *C. elegans*.

#### 2.2.1. Daf-16/FOXO

**Figure 3:**
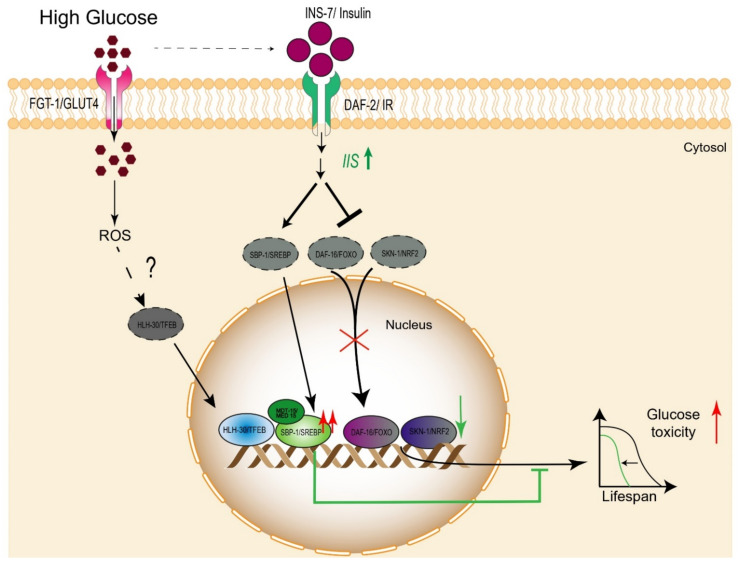
Dietary glucose decreases lifespan by altering cellular localization of several transcriptional factors. High glucose, probably through activation of the IIS pathway, diminishes the nuclear localization and transcriptional activity of DAF-16/FOXO and SKN-1, which consequently decreases the worms’ lifespan. Interestingly, high glucose augments the nuclear localization of HLH-30 and SBP-1/MDT-15, although with opposite consequences, because HLH-30 decreases the lifespan whereas SBP-1/MDT-15 prevents the life-shortening effects of high glucose.

FOXOs (forkhead box O) transcription factors are involved in the regulation of lifespan and longevity via insulin and insulin-like growth factor signaling through the modulation of multiple cellular pathways that regulate several biological processes in mammals [39]. Some studies have associated the possible role of FOXO factors with human longevity; specifically, several alleles from the FOXO1A and FOXO3A genes are enriched in various centenarians populations [40,41,42], while other studies have indicated that FoxO3 is required for the lifespan extension induced by caloric restriction in mice [43]; however, how these factors mediate these effects remains to be fully elucidated. Interestingly, in cardiac microvascular endothelial cells, high glucose stimulates ROS production and inhibits the protein kinase B (PKB/AKT), which subsequently activates FOXO3A and apoptosis [44]. Additionally, it has been documented that FOXO also plays an important role in adverse effects on systemic glucose intolerance and hepatic insulin resistance; therefore, targeting these factors could be an effective strategy for the treatment of diabetes mellitus in patients with hepatic insulin resistance [45,46]. In a similar way, the *C. elegans* FOXO homolog DAF-16 regulates and integrates diverse pathways responsible for stress resistance and immune response, although it is notorious for its role in the lifespan mediated by the insulin-PI3K-AKT pathway [47,48,49]. *C. elegans* has several DAF-16 isoforms: DAF-16 a, b, d, and f, which are expressed in different tissues and functions, although DAF-16 a and f appear to have more important roles in lifespan [50]. DAF-16 activation relies on post-translational regulation, whereby its translocation into the nucleus is mediated by its phosphorylation status, driven mainly by the insulin–insulin-like growth factor 1 (IGF-1) signaling (IIS) pathway and nutritional state. Specifically, any conditions that reduce IIS or nutrient scarcity lead to DAF-16 nuclear translocation, whereby it modulates transcription of its target genes, promoting lifespan extension in worms (Table 1). Several studies have documented that a glucose-enriched diet significantly shortens the lifespan of *C. elegans*. Interestingly, it appears that DAF-16 may play a role in regulating this effect. Nematodes grown on a highly glucose-enriched diet showed decreased activity of DAF-16, which occurred through activation of its principal negative regulator, the IIS [51]. This fact was proven, because under a high-glucose diet, the mutant strain of DAF-16 did not further decrease its already reduced lifespan, and interestingly it can generate a genomic response similar to wild-type nematodes grown under a high-glucose diet; similarly, a long-lived mutant of the insulin receptor (*daf-2*) with reduced IIS also exhibited a short lifespan when exposed to a high-glucose diet (Table 1) [51]. As such, high glucose intake stimulates insulin signaling, which decreases lifespan in part by inhibiting DAF-16 (Figure 3).

Interestingly, the treatment of worms with the phospholipid phosphatidylserine promotes lifespan extension and requires the presence of DAF-16 to mediate its effects. Given that phosphatidylserine may restore the reduced lifespan caused by exposure to a high-glucose diet, it could be an outcome induced by DAF-16 [52].

Conversely, opposite conditions, such as starvation, lead to the activation of DAF-16, which promotes metabolic enzymes, reprograms carbohydrate metabolism, and shifts the carbon flux toward the glyoxylate shunt and gluconeogenesis, causing trehalose synthesis and catabolism to generate energy, which supports the survival of *C. elegans* [53].

Others have demonstrated that DAF-16 in a high-glucose diet can prolong the lifespan by regulating multiple antioxidants and chaperones, as well as immunity, metabolic, and other genes that act in a cumulative way to positively influence the lifespan [51,54]. For example, the glycerol channels in glucose metabolism *aqp-1* were also a glucose-regulated downstream target of DAF-16 [51]. AQP-1 is a glycerol channel; it is also possible that glycerol itself is an intercellular signal that can act on other tissues to influence the expression of DAF-16 target genes.

**Table 1:** List of genes that alter the lifespan in *C. elegans* and mammals.

*C. elegans* Gene	Human Homologue	Gene Description	Effect on Lifespan		References
			Gain of Function	Loss of Function	
*daf-2*	INSR	Insulin receptor	ND	Increase ^a,b^	[55,56]
*fgt-1*	SLC2A4	Solute Carrier Family 2 Member 4	ND	Increase ^a^	[27]
*daf-16*	FOXO	Forkhead Box O1	Increase ^a^	Decrease ^a^	[53,57]
*skn-1*	Nrf2	Nuclear factor, erythroid 2-like 2	Increase ^a,b^	Decrease ^a^	[58,59,60,61]
*hlh-30*	TFEB	Transcription factor EB	Increase ^a^	Decrease ^a^	[62]
*sbp-1*	SREBP	Sterol Regulatory Element Binding Transcription Factor	Increase ^a,b^	Decrease ^a^	[63]
*mdt-15*	MED15	Mediator Complex Subunit 15	Increase ^a^	ND	[64]
*nhr-49*	PPARα	Peroxisome Proliferator Activated Receptor Alpha	Increase ^a^	Decrease ^a^	[65]
*nhr-80*	HNF4A	Hepatocyte Nuclear Factor 4 Alpha	Increase ^a^	Decrease ^a^	[66,67]
*xbp-1*	XBP1	X-Box-Binding Protein 1	Increase ^a^	Decrease ^a^	[68]

^a^ = *C. elegans*, ^b^ = mammals, ND = Not determined.

#### 2.2.2. SKN-1/Nrf

It is well documented that overconsumption of glucose may increase cellular reactive oxygen species (ROS) levels, which not only negatively impact glucose metabolism but dysregulate cellular signaling and a variety of genetic pathways, thereby causing apoptosis and cell death [69,70]. The mammalian Nrf/CNC proteins perform a wide range of cellular protective and maintenance functions, with Nrf2 being the key regulator of antioxidant and xenobiotic defense, in addition to contributing to proteostasis and metabolic regulation [71]. In this sense, it has been suggested that the Nrf2 transcription factor is an essential modulator of longevity. Studies from long-lived rodent species, such as the naked mole rat, showed that this animal had markedly higher levels of signaling activity of Nrf2 compared with short-lived mice, probably because an increase in Nrf2 signaling helps them to combat inevitable endogenous stressors, such as ROS, thereby promoting increased lifespan and longevity [58]. Consistent with Nrf2’s function, its overexpression in cell cultures grown under high glucose intake reduces its deleterious effects, thereby promoting cell survival; therefore, Nrf2 plays a critical role in cellular protection against metabolic insults [72]. The transcription factor SKN-1, the *C. elegans* ortholog of mammalian Nrf protein, plays an important role in oxidative stress defense and longevity [59,60]. ROS produced from bacterial and fungal pathogens trigger increased expression of genes that encode several antioxidant and stress-protective enzymes and are mediated by the SKN-1 transcription factor [73]. Studies related to worms fed with a high-glucose diet showed that nematodes with constitutively activated SKN-1 avoid an increased lipid accumulation phenotype compared with wild-type worms, suggesting that prolonged SKN-1 activation promotes a metabolic remodeling that predisposes animals to successfully cope with these dietary insults [74]. Concerning lifespan, the data indicate that high glucose inhibits SKN-1 by increasing IIS pathway activity and blocking the pro-longevity effects conferred by translation initiation factor eIF4F (Figure 3) [37,75]. It is well known that high glucose levels negatively affect immune response, and since SKN-1 is an important regulator of innate immunity, Li et al. exposed nematodes to infection with Salmonella typhimurium and high glucose; then, they evaluated the effects on SKN-1 and found that nematodes grown in high glucose and previously infected with *S. Typhimurium* had shorter survival times, in part due to the inhibition of the SKN-1 pathway. Conversely, hyperactivation of SKN-1 by diminishing its negative regulator WDR-23, a homolog of mammalian KEAP-1, reverses the negative effects of glucose and restores the lifespan (Table 1) [61].

#### 2.2.3. HLH-30/TFEB

Autophagy is a conserved cellular degradation process that maintains a non-toxic environment under normal conditions by degrading and recycling misfolded proteins and damaged organelles; nevertheless, it is also vital for metabolic homeostasis in organisms under stressful conditions by providing nutrients and molecular building blocks, and is considered to positively regulate longevity [76]. One of the primary transcriptional regulators of autophagy in mammals is the transcription factor EB (TFEB). TFEB is considered the master regulator of lysosomal biogenesis and autophagy, and its activation has been proven to drive cell and organism benefits [77]. Although TFEB has been recognized as a pro-survival transcription factor, it has been shown that TFEB activation can promote adverse effects. Tseng et al. found that monocytic cells exposed to high glucose (30 mM) induced release of lysosomal Ca^2+^, thereby increasing cytosolic Ca^2+^ and promoting TFEB nuclear translocation, possibly through the activation of the calcium-dependent phosphatase calcineurin, a phosphatase that activates TFEB. The authors also showed that nuclear TFEB favored lysosomal-exocytosis-mediated IL-β secretion, a pro-inflammatory cytokine that has been related to impair insulin secretion and shown to contribute to chronic inflammation reported in type 2 diabetes mellitus patients [78,79], strengthening the idea that although there is evidence that TFEB activation can promote a response that helps the cell to deal with stress, under specific circumstances it could trigger detrimental effects. The *C. elegans* TFEB ortholog is HLH-30 [80,81]. HLH-30/TFEB is predominantly localized in the cytoplasm under basal conditions and translocates into the nucleus upon several cellular stresses, such as lysosomal impairment, bacterial infection, prolonged endoplasmic reticulum (ER) stress, reactive oxygen species (ROS), and nutrient scarcity [82,83,84,85]. It has been shown that the activation of HLH-30/TFEB during nutrient deprivation leads to an increase in the lifespan of *C. elegans* (Table 1) [62]; however, recent reports have shown that HLH-30/TFEB, similar to mammals, could respond in an unexpected manner depending on the type of stimulus, such as elevated ROS levels [84,86]. As such, depending on the cellular context, HLH-30 might lead to cell death, suggesting that HLH-30/TFEB has a new role as a potent inducer of cell death in different stress conditions that have not been studied extensively thus far. Consistent with this idea, Franco-Juarez et al. exposed nematodes to a high-glucose diet and found that nematodes showed increased autophagic flux via an HLH-30/TFEB-dependent mechanism; however, their lifespan was diminished (Figure 3). Since the nuclear localization of HLH-30/TFEB is dependent on its phosphorylation, they also treated worms with okadaic acid, an inhibitor of the PP2A and PP1 protein phosphatases, and found that this acid was capable of blocking the HLH-30/TFEB nuclear translocation during glucose exposure, suggesting that HLH-30/TFEB translocation is dependent on its phosphorylation status and might have detrimental effects on lifespan, possibly through autophagy under this stress condition [87]. In line with these outcomes, Mellor et al. found that mice fed with a high fructose diet increase myocardial ROS production; moreover, they reported an increase in autophagy, perhaps as a consequence of elevated ROS, because ROS had been associated with induction of autophagy as part of the cell mechanism to protect cells from apoptosis. Nevertheless, it has been reported that excessive activation of autophagy can trigger non-apoptotic cell death. Interestingly, they also suggested that the insulin resistance induced by a high-fructose diet could be a consequence of autophagy activation; therefore, autophagy can act as a double-edged sword, and similarly to TFEB/HLH-30, it can trigger unexpected cell responses [88,89,90,91]. Although Mellor and colleagues did not show that activated autophagy was dependent on activated TFEB, it could be possible that autophagy could be triggered by TFEB, as ROS had been reported to induce TFEB nuclear translocation. Nevertheless, further studies need to be performed to elucidate whether autophagy activation under high-fructose diet is mediated by the ROS-TFEB axis.

Mammalian autophagy is regulated negatively by the serine–threonine kinase mTOR, by phosphorylation and inhibition of initial autophagy proteins, and by phosphorylation of TFEB [92,93]. The mTOR pathway is activated by hormones, as well as growth factors, such as glucose, amino acids, and fatty acids, thereby inhibiting catabolic pathways such as autophagy; although it has been shown that inhibition of TOR signaling in *C. elegans* after development induces lifespan extension and that pharmacological inhibition of mTOR induces autophagy promoting cell survival, nonetheless many reports suggest that TFEB and autophagy can be activated independently of mTOR activity, triggering unforeseen responses as a mechanism to cope with cell stress [94,95,96,97].

#### 2.2.4. SBP-1/SREBP

Large amounts of dietary carbohydrates such as glucose are stored as lipids; therefore, glucose and fat metabolism are tightly related. Moreover, cellular disruption of lipid and cholesterol metabolism results in pathological processes linked to metabolic and cardiovascular diseases. Sterol regulatory element-binding proteins (SREBPs) are key transcription factors that modulate fat metabolism by controlling several lipogenic genes [98,99]. The levels of SREBP-1 are significantly elevated in obese patients and animal models of obesity and type 2 diabetes; however, it appears that the contribution of SREBP-1 to improving insulin resistance is poor, because even in the absence of SREBP-1 elevated glucose levels persist. Caloric restriction is a condition that has been shown to extend the lifespan of organisms across the evolutionary spectrum, whereby SREBP is involved because of enhancement of fatty acid biosynthesis and mitochondrial activation, resulting in augmentation of the organismal lifespan [63]. In addition, the grown of Schwann cells in high glucose showed a significant diminution of SREBP-1 via the blockage of the AKT signaling pathway [100]. In *C. elegans*, SBP-1, the transcription factor SREBP ortholog, induces lipid synthesis together with the transcriptional coregulator mediator-15 (MDT-15) by activating the expression of lipogenic genes, in addition to regulating lifespan in nematodes [64,101,102,103]. It has been documented that worms exposed to high glucose have a reduced lifespan, in part due to the accumulation of saturated fat. Interestingly, Lee et al. found that the deleterious effects of glucose arose due to the accumulation of saturated fat; however, overexpression of SREBP and MDT-15 substantially prevented the life-shortening effects of high glucose; thus, both regulators protect *C. elegans* from the toxic effects of dietary glucose on lifespan by promoting the conversion of saturated fatty acids to unsaturated fatty acids (Table 1, Figure 3) [38]. Finally, it was found that the phosphatidic acid phosphatase, lipin-1/LPIN-1, also prevents the life-shortening effects of dietary glucose. In fact, genetic depletion of lpin-1 results in an increased fatty acid synthesis and desaturase gene and maintains proper lipid homeostasis. Furthermore, these changes in gene expression appear to be caused by the activation of SBP-1 in *lpin-1* RNAi-treated animals; however, some future directions for research include studying the relationship between SBP-1 and LPIN-1 within the context of lifespan [104].

### 2.3. Fructose

Fructose is a monosaccharide that is generally found in fruits, some vegetables, and honey, and is a common sweetener typically found in the form of high-fructose corn syrup. Similar to glucose and other sugars, chronic fructose consumption may trigger several metabolic diseases such as metabolic syndrome, obesity, fatty liver, hypertension, and diabetes [105,106]. It has been shown that high-fructose or -sucrose diets promote insulin resistance and aggravate Alzheimer’s disease and related pathologies [107]. Fructose is mostly absorbed in the small intestine via the hexose transporter SLC2A5 (GLUT5), which is a key regulator of the plasma concentration of fructose, and is then metabolized by the liver, which is considered the main organ for fructose metabolism [108]. Unlike glucose breakdown, fructose is a weak substrate for hepatic glucokinase; therefore, its catabolism is carried by ketohexokinase (KHK), which phosphorylates fructose to generate fructose-1-phosphate (F1P) [109]. F1P is metabolized to dihydroxyacetone phosphate (DHAP) and glyceraldehyde 3-phosphate (G3P), which can enter gluconeogenesis or be metabolized into lactate or acetyl-CoA, which is oxidized or used for de novo lipogenesis. As such, fructose has effects on both glucose levels and lipogenesis; thus, excessive fructose intake has detrimental effects on human health [110].

Although it has been described that the consumption of a fructose-rich diet may stimulate dyslipidemia and impair the effects of insulin on hepatic glucose production in humans [111], the precise mechanisms responsible for this remain unknown. Studies based on animal models have demonstrated that the addition of fructose to drinking water or solid foods leads to energy overconsumption, which triggers the development of obesity, insulin resistance, dyslipidemia, and hepatic steatosis, consequently leading to decreased life expectancy [112,113,114]. Additional studies have shown that female mice fed with a high-fructose or -glucose diet showed an almost two-fold increase in mortality, and that these diets decreased the reproductive success of male mice; therefore, an increase in fructose consumption not only promotes metabolic disorders but also contributes to lifespan and healthspan decreases [115].

Studies in *C. elegans* showed that the effect of fructose on lifespan is concentration-dependent. Zheng et al. found that nematodes grown with 55 and 111 mM of fructose increased their lifespan by 22% and 45.7%, whereas increased fructose concentration by 10 times (555 mM) had an opposite effect, since lifespan was reduced [116]. Similar results were obtained by Wang et al., suggesting that the lifespan of *C. elegans* is related to the concentration of ingested fructose [117]. Consistent with the above studies, Chen et al. exposed nematodes to different fructose concentrations, such as 5% (278 mM), 10% (555 mM), and 15% (833 mM), and evaluated lifespan, growth, and fertility for 6 days. The study showed that from the first day with lower fructose doses (5%), the lifespan and fertility were better than with higher fructose doses, because at 10 and 15% doses of fructose, the population decreased by around 50%, suggesting that higher fructose doses alter *C. elegans*’ metabolism, leading to increased fat accumulation. Similarly to others, they proposed that the direct amount of fat deposition is harmful to the healthspan and leads to the death of *C. elegans*. Despite the results mentioned above suggesting that the effects of fructose on the lifespan of *C. elegans* are due to a high fructose concentration (555 mM), a recent study [118] found that at chronic low-fructose concentrations (5%), wild-type nematodes showed impaired healthspans, as observed by low movement, low food intake, and decreased lifespan. These differences were probably due to the length of exposure to fructose, although more studies are necessary to clarify these differences.

### 2.4. Trehalose

Trehalose is a disaccharide of glucose and is synthesized by a wide variety of organisms ranging from prokaryotes to eukaryotes, such as fungi and invertebrates, with significant metabolic roles, including storage of energy; however, it is not present in mammals [119]. Unlike other sugars, trehalose has lower sweetener properties and is metabolized through the trehalase enzyme into two glucose molecules to provide a high amount of energy [120]. In humans, it has been shown that trehalose may modulate glucose homeostasis in multiple tissue types, in part by activating fasting signaling, which consequently reduces hepatic steatosis and activates adipose tissue browning. Additionally, it has been shown that trehalose treatment in humans may reduce cardiometabolic disease, hepatic steatosis, and insulin resistance; however, the mechanisms by which trehalose exerts its beneficial effects are not well understood [121]. In most organisms, including nematodes, trehalose synthesis involves the following steps. First, the enzyme trehalose-6-phosphate synthase (TPS) catalyzes the transfer of glucose from UDP-glucose to glucose-6-phosphate to produce the intermediate molecule trehalose-6-phosphate (T6P). Then, trehalose-6-phosphate phosphatase (TPP) dephosphorylates T6P to form trehalose [119,122]. In *C. elegans*, three genes (*tps-1*, *tps-2*, and *gob-1*) encode for enzymes that mediate trehalose synthesis [123]. Two are trehalose phosphate synthase enzymes, (TPS-1 and TPS-2) while one is trehalose-6-phosphate phosphatase (*gob-1*), and the respective gene expression occurs at all stages of *C. elegans’* lifecycle [124]. Similarly to other invertebrate species, it has been proposed that trehalose also has important physiological functions in *C. elegans*, since this disaccharide may be used as an energy source, stress protectant, and inducer of an increased lifespan. Consistent with the proposed role of *tps* genes, RNA interference (RNAi) targeting of both *TPS1* and *TPS2* in wild-type animals resulted in a >90% decline in trehalose levels; however, no obvious phenotype was observed. Conversely, TPP (*gob-1*, gene) knockdown enzyme in *C. elegans* resulted in lethal phenotypes, which was completely suppressed by the ablation of *C. elegans tps-1* and *tps-2* genes [124].

The IIS mutant daf-2, bearing a mutation in the insulin/IGF-1 receptor, is characterized by living about twice as long as the wild type and having about five-fold higher trehalose levels compared with the wild type [125]. Since trehalose function is not only circumscribed as a reserve carbohydrate but also as an important stress-protecting molecule in different organisms, Honda et al. analyzed the effect of adding trehalose to the culture medium of *C. elegans* and found that 5 mM of trehalose extended the lifespan and thermotolerance and retarded aging independently of age state, apparently since trehalose prevents the formation of protein aggregates that normally accumulate with age [126]. Interestingly, a similar finding was obtained by Seo et al., whereby animals fed with a diet supplemented with 5 mM trehalose showed increased internal trehalose and lifespan extension. Moreover, these positive effects were in part dependent on the transcription factor DAF-16 and the autophagy process, because trehalose activates *pha-4*, a transcription factor that activates genes for the synthesis and maturation of the autophagosome [31]. In summary, high levels of trehalose in *C. elegans* improve healthy aging through DAF-16 and autophagy [31].

## 3. Effects of Lipids on Lifespan

Many of the genetic and signaling pathways that are related to lipid metabolism are linked to lifespan [127]. Fatty acids are structurally varied molecules that can be obtained from dietary intake and de novo synthesis, having diverse biological functions. It is known that with age there are changes in the metabolism of lipids, which include alterations in their transport, synthesis, and breakdown; therefore, recent findings have shown that age-related diseases could be a consequence of alterations in lipid composition. Nevertheless, the mechanisms that promote such changes have not been completely delineated [128,129]. Studies derived from different animal models of exceptional longevity, such as the naked mole rat, suggest an association between lipid composition and animal longevity. These organisms present a specific fatty acid saturation profile that probably helps maintain membrane fluidity by preventing peroxidation, the preservation of membrane lipid composition, which may be a key component of their increased longevity [130].

### 3.1. PUFAs

Polyunsaturated fatty acids (PUFAs) are a group of biomolecules that contain more than one double bond in their molecular structure and are considered to be important nutrients that regulate many physiological processes. Since they are major structural cell membrane components that participate in cell signaling pathways, it has been proposed that PUFAs may prevent some diseases, such as cancer, diabetes, cardiovascular disease, and Alzheimer’s disease [131,132]. There is substantial evidence in experimental models implicating PUFAs in neuroinflammatory, neurotrophic, neuroprotective, and cognition processes in the brain [133]. The majors PUFA groups are omega-3 (n-3) and omega-6 (n-6). PUFAs are named after the position of the first double bond from the methyl end in a fatty acid chain [134,135]. In mammals, PUFAs are synthesized through fatty acid desaturases (FADSs), which introduce double bonds (desaturation) into the aliphatic chain of fatty acids and the extension (elongation) via two carbon units of the acyl chain; therefore, the biosynthetic pathway involves the alternate actions of desaturase and elongase enzymes [136]. Although lower amounts of eukaryotes may produce omega-3 and omega-6 fatty acids, mammals, including humans, cannot synthesize omega fatty acids de novo and must obtain them from the diet.

Several studies have shown that ω-3 PUFAs possess a therapeutic role in human disease because they improve coronary heart disease, reduce microinfarct burdens, and have significant implications in neuronal processes that contribute to language ability and communication fluency, among others; conversely, ω-6 PUFAs have been associated with a proinflammatory response that may lead to the development of several diseases, such as nonalcoholic fatty liver disease, cardiovascular disease, Alzheimer’s disease, and cancer [137,138,139]. PUFAs are most susceptible to lipid peroxidation. It has been suggested that organisms whose cellular membranes contain high amounts of PUFAS have shorter lifespans due to increased susceptibility of the membranes to lipid peroxidation; however, other studies do not support this hypothesis, since it has been found that dietary supplementation of PUFAS does not affect the lifespan of mice [140].

Similarly to all life forms, the regulation of lipid metabolism in *C. elegans* is influenced by the environment. The temperature, nutrient availability, and physiological state, including growth, reproduction, development, and aging, play essential roles in survival [141]. *C. elegans* can synthesize wide range of PUFAs de novo a because this organism presents all of the enzymes necessary for this function, including acetyl CoA carboxylase (*acc1,2*), fatty acid synthase (*fasn-1*), fatty acid desaturase (*fat-1*, *fat-2*, *fat-3*, and *fat-4*), and elongase activities (*elo-6*), allowing *C. elegans* to synthesize long-chain PUFAs such as arachidonic acid (AA, 20:4) and eicosapentaenoic acid (EPA, 20:5) [14].

**Figure 4:**
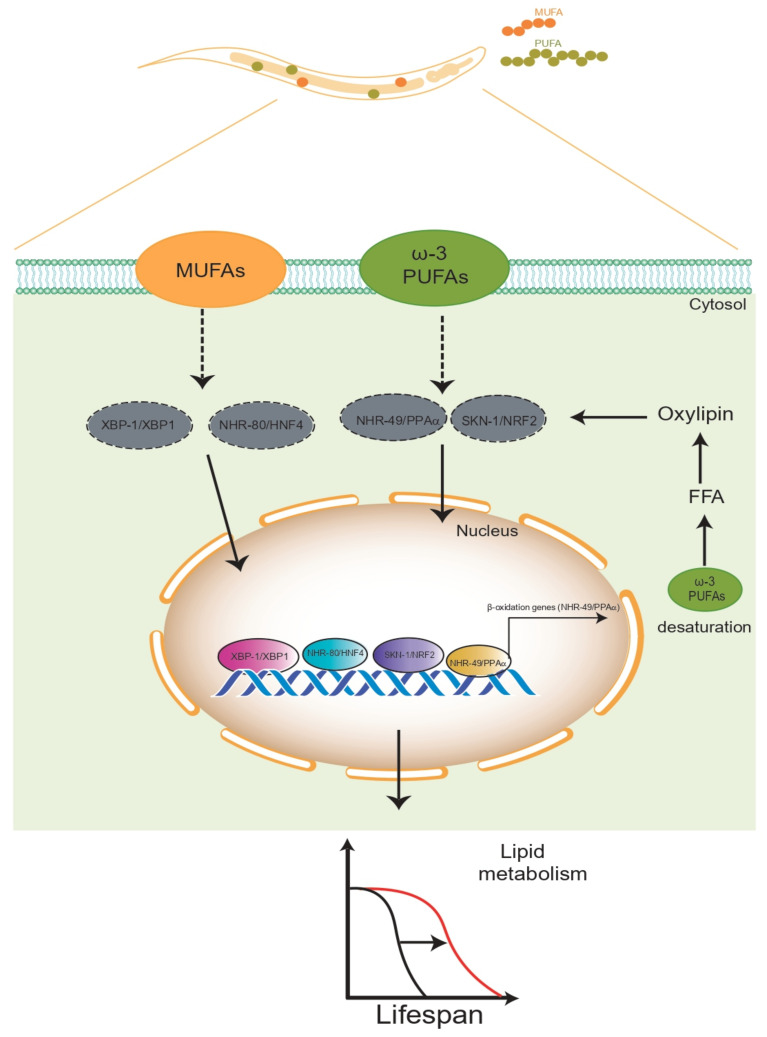
Effects of different dietary lipids on the lifespan of *C. elegans*. Exogenous MUFAs and PUFAs contribute to lifespan extension via activation of several transcriptional factors that promote gene transcription of enzymes for fatty acid desaturation, which might play an important role in the positive regulation of the lifespan.

Several studies leading to a better understanding of the effect of ω-6 PUFAs in the cellular and physiological processes of *C. elegans* found opposite specific effects, since supplementation with the ω-6 PUFA di-homo-γ-linoleic acid (DGLA, 20:3n-6) in wild-type nematodes had detrimental effects on the reproductive system, as the DGLA treatment led to sterility due to a lack of germ cells, which increased in a dose-dependent manner; contrarily, DGLA and AA supplementation enhanced the ability to resist starvation, suggesting that these ω-6 PUFAs promote *C. elegans’* survival during starvation. Moreover, the treatment with AA and DGLA increased *C. elegans’* lifespan through the activation of autophagy in well-fed conditions [142]. Interestingly, several studies have shown that long-lived *glp-1* germ line-less mutant animals were correlated with extended lifespans and with increased production of the ω-3 fatty acid, α-linolenic acid (ALA). Based on these observations, Qi et al. showed that treating *C. elegans* with different ALA concentrations (ranging from 2 to 10 mM) significantly increased the lifespan via the activation of the NHR-49/PPARα and SKN-1/Nrf2 transcription factors, whereby NHR-49 promotes the expression of genes involved in the β-oxidation of lipids, whereas SKN-1 was activated by ALA-derived oxylipins that promote increased longevity resulting from ALA treatment (Table 1, Figure 4) [66]. On other hand, supplementation with ω-3 fatty acids, such as linoleic acid (LA, 18:2n-6) and eicosapentaenoic acid (EPA, 20:5n-3), had no side effects on the worms, as seen with ω-6 DGLA [143].

### 3.2. MUFAS

Monounsaturated fatty acids (MUFAs) are fatty acids that contain a single double bond. As with other lipids, MUFAS can be acquired exogenously (from the diet) or endogenously synthesized; for mammals, their anabolism occurs in the liver, with the adipose tissue, through stearoyl-CoA desaturases (SCDs), from their saturated fatty acid–acetyl-coenzyme A precursors, forming MUFAs. SCDs are the rate-limiting enzymes for MUFA formation [144]. Briefly, SCDs introduce a delta-9 desaturation in the SFAs stearate (C18:0) and palmitate (C16:0), forming the MUFAs oleate (C18:1n-9) and palmitoleate (C16:1n-7), respectively. MUFAS in *C. elegans* can be obtained from de novo synthesis through saturated fatty acid precursors via desaturation or bacterial membrane digestion. In particular, several studies suggest important roles for specific MUFAs (oleic acid, OA) in increasing lifespan [67,102]. In fact, nematodes lacking the germline (*glp-1*, *e2141ts* strain) have an increased lifespan due to a mechanism that is implicated in the activation of fatty acid desaturase, FAT-6/SCD1, the expression of which is dependent on the nuclear receptor NHR-80/HNF4, which promotes the monodesaturation of stearic acid to OA (Table 1) [67]. Oleic acid seems to be implied in extending the lifespan, since metabolomic analysis on the long-lived mutant *daf-2* (e1370 strain) indicates higher concentrations of OA compared to wild-type worms [145]; this is in line with the findings of Imanikia et al., who found that nematodes overexpressing XBP-1, a transcription factor that has been recently proposed to regulate glucose and lipid metabolism in mammals, have increased levels of OA and a concomitant augmented lifespan (Table 1, Figure 4). Notably, the worms with overexpression of XBP-1 show increased transcription of desaturase FAT-6, the delta-9 desaturase that turns stearic acid into OA [68]. Moreover, other studies showed that the addition of 2 mM OA to nematode growth medium (NGM) plates was sufficient to consistently and significantly increase the lifespan of wild-type worms (Figure 4) [144]. Finally, studies related to the COMPASS family of H3K4 methylases showed that deficiency in H3K4me3 methyltransferase in *C. elegans* promotes specific enrichment of MUFAs, and based on these data, other researchers found that dietary supplementation of individual MUFAs (oleic, palmitoleic, or cis-vaccenic acid) was sufficient to extend the lifespan of the wild-type strain, conceivably by promoting membrane fluidity, minimizing oxidative stress, enhancing energy storage, or activating pro-survival signaling pathways [102].

## 4. Pharmacologic Strategies That Extend the Organismal Lifespan

**Figure 5:**
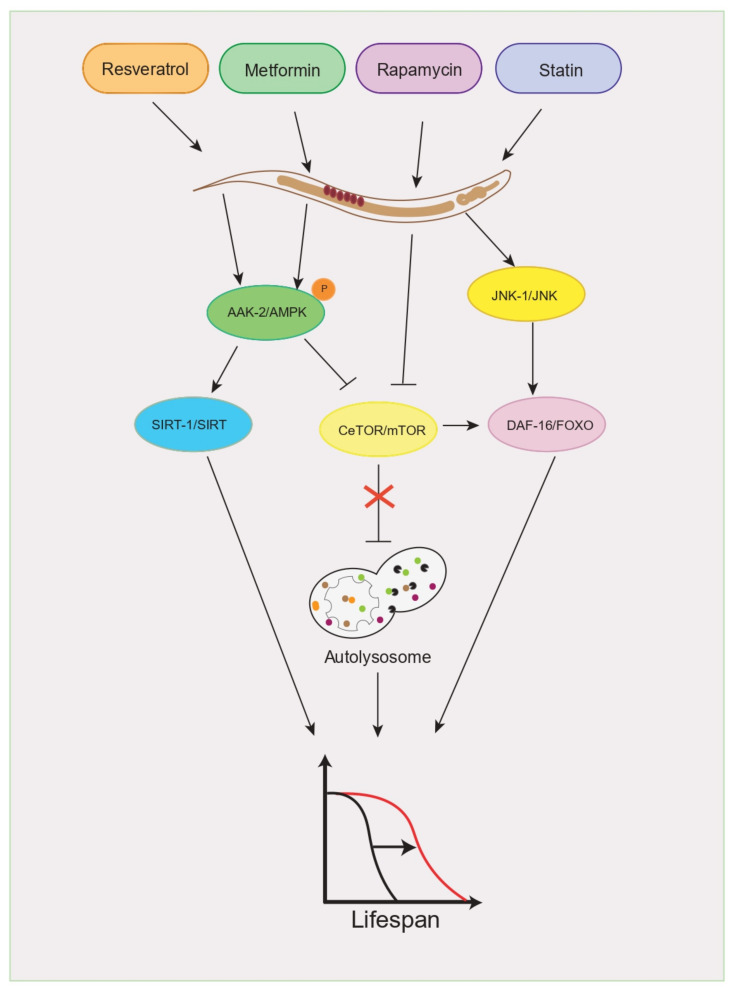
The schematic represents a simplified overview of the signaling pathways regulating organismal lifespan under the influence of drugs.

Emerging data suggest that carbohydrate–lipid intake plays a critical role in metabolic dysregulation that impacts longevity. A pharmacological treatment approach can be used in the treatment of metabolic diseases. Several drugs have been shown to improve lifespan when affected by energetic sources. Metformin is a drug commonly prescribed to treat patients with type 2 diabetes and obesity, the benefic effects of which enhance insulin sensitivity, induce glycolysis, and suppress gluconeogenesis in the liver [146]. It has been reported that chronic metformin exposure lengthens the lifespan and attenuates the deleterious effects of aging in male mice. The effects of metformin to some extent resemble the effects of caloric restriction, even if food intake is increased [147]. Interestingly, as in mammals, metformin promotes health and extends the lifespan in *C. elegans*, whose mechanism of action is attributed to the activation of adenosine-monophosphate-activated protein kinase (AAK-2/AMPK), which inhibits mTORC1 and consequently activates autophagy and the lysosomal pathway (Figure 5) [148,149,150,151]. Statins are among the most prescribed drugs worldwide to treat hypercholesterolemia. They specifically inhibit the enzyme hydroxymethylglutaryl coenzyme A (HMG-CoA) reductase, thereby preventing the conversion of HMG-CoA to mevalonate, which represents an early rate-limiting step in cholesterol biosynthesis [152]. Statin drugs have been studied in a large number of individuals, showing that these drugs reduce the risk of atherosclerotic cardiovascular disease and mortality with few side effects [153]. Analysis of the effects of statins (lovastatin), as well as genetic inhibition of the HMG-CoA reductase in *C. elegans*, showed that low doses of lovastatin prolong its lifespan via induction of Jun N-terminal kinase (JNK-1), a known activator of DAF-16/FOXO, as well as some DAF-16 target genes related to lifespan extension (Figure 5) [154]. Resveratrol (3,5,4′-trihydroxystilbene) is a polyphenol abundant in red grapes. The treatment with this compound has been shown to prevent the deleterious effects of excess caloric intake through modulation of energy balance. Specifically, resveratrol shifts the physiology of mice consuming excess calories via increased insulin sensitivity, reduced insulin-like growth factor-1 (IGF-I) levels, increased AMPK, and peroxisome-proliferator-activated receptor-γ coactivator 1α (PGC-1α) activity, which consequently increases mitochondrial quantity and improves motor function [155]. The mechanism of action of resveratrol remains elusive; however, AMPK and the NAD-dependent deacetylase SIRT1 have been proposed to mediate the pro-survival response. The pro-longevity effects of resveratrol in *C. elegans* are conveyed through sirtuin-1-dependent induction of autophagy, suggesting that this process is universally required for the lifespan-prolonging effects of pharmacological sirtuin-1 activators (Figure 5) [156]. Although resveratrol exhibits certain benefits on lifespan, these findings were questioned by a report suggesting that resveratrol supplementation only marginally increases the lifespan of *C. elegans* and that this small effect is preserved in Sir2-mutant strains [157]; thus, future studies will need to be carefully designed to examine the effects of resveratrol on the organismal lifespan. Rapamycin is an antifungal metabolite that is well known for its inhibitory effects on mTOR in mammals [158]; mTOR signaling is an important player in longevity regulation. In mammals, rapamycin extends the median and maximal lifespans of both male and female mice when feeding begins at 1.6 years of age [159]. Acute or chronic treatment with rapamycin abrogates insulin resistance in mice; however, it has also been reported that chronic treatment with rapamycin can also cause glucose intolerance and even insulin resistance [160,161]. Genetic or pharmacological (rapamycin) inhibition of mTOR signaling has been found to extend the lifespan of *C. elegans*. When TORC1 is inhibited, either genetically or with rapamycin, *C. elegans* triggers activation of SKN-1 and DAF-16 to promote several protective genes, increasing stress resistance and longevity (Figure 5) [162].

## 5. Conclusions

Malnutrition and lifestyle are critical factors that promote metabolic diseases, which negatively impact upon health and longevity; therefore, a better understanding of the underlying biological mechanisms is relevant to human health. Here, we review various cell signaling pathways and the effects of dietary nutrients, which can lead to detrimental effects on longevity and lifespan and can influence aging. We also highlight the advantages of *C. elegans* as an alternative organism that models the effects of nutrition on health and lifespan. Knowledge about the interactions of pathways controlling genomic responses to dietary interventions can provide strategies that can be used to elucidate potential therapeutic avenues against metabolic disorders and age-related diseases and to counteract their impacts on lifespan.

## Data Availability

Not applicable.
